# High resolution spiral myocardial phase velocity mapping (PVM) of the entire cardiac cycle

**DOI:** 10.1186/1532-429X-15-S1-O6

**Published:** 2013-01-30

**Authors:** Robin Simpson, Jennifer Keegan, David N Firmin

**Affiliations:** 1 NIHR Cardiovascular Biomedical Research Unit, Royal Brompton Hospital, London, UK; 2 Imperial College, London, UK

## Background

Three-directional PVM is capable of measuring regional myocardial velocities. Current techniques use Cartesian k-space coverage, and navigator-gated high spatial and temporal resolution acquisitions are long [1,2]. In addition, they use prospective ECG-gating and analysis of the full cardiac cycle is not possible. The aim of this study is to develop a high temporal and spatial resolution PVM technique using efficient spiral k-space coverage and retrospective ECG-gating which will allow detailed analysis of the entire cardiac cycle, including atrial systole which accounts for 20-30% of left-ventricular filling in healthy motion [3].

## Methods

K-space is covered with 13 spiral interleaves (12ms duration, TR 21ms). Navigator-gated reference and 3-directional velocity-encoded data (15cm/s in-plane, 25cm/s through-plane) are acquired in consecutive cardiac cycles following a single dummy cycle (nominal duration 53 cardiac cycles). The acquired spatial resolution is 1.4x1.4x8mm (reconstructed to 0.7x0.7mm). Retrospective gating allows full coverage of the cardiac cycle with 60 phases per RR-interval (reconstructed temporal resolution 14-20ms depending on heart-rate). Basal, mid and apical short-axis slices were acquired in 10 healthy volunteers on a Siemens Skyra 3Tesla scanner. Radial, circumferential and longitudinal velocities were extracted and early systolic, early diastolic and late diastolic (atrial systole) peak velocities and times to those peak (TTP) velocities were measured.

## Results

**Figure 1:**
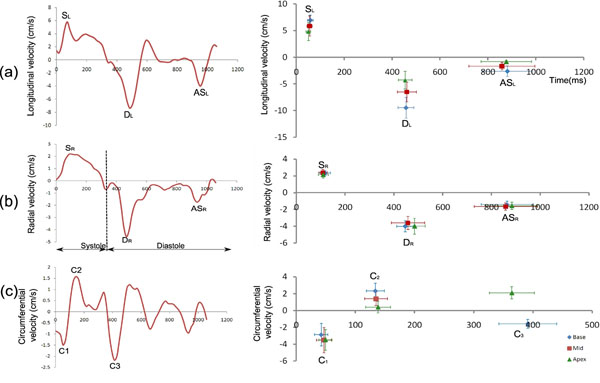
Example velocity time curves (left column) and mean +/- SD peak velocities and TTP values (right column) for longitudinal (a), radial (b) and circumferential (c) directions. End systole is marked by a vertical dotted line on the radial curve. The main peaks (systole (S), early diastole (D) and atrial systole (AS) for longitudinal and radial velocities and the three circumferential peaks (C1, C2 and C3)) are marked. Of particular note is the late diastolic peak which has not previously been seen with prospectively gated PVM studies and which occurs due to passive movement as the atria contract, producing a velocity peak in the radial and longitudinal directions.

**Figure 2:**
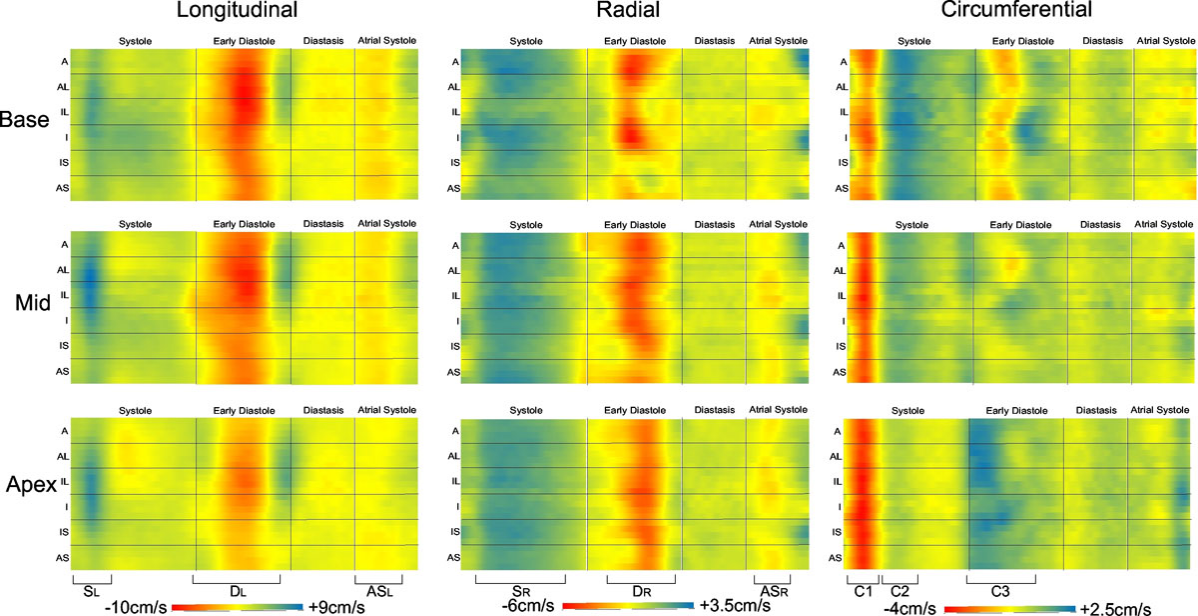
Basal (top), mid (middle) and apical (bottom) short-axis colour-maps displaying regional longitudinal (left), radial (middle) and circumferential (right) velocities (from anterior wall through to lateral, inferior and septal walls on the vertical axis) against time after the R-wave (horizontal axis), averaged over the ten healthy volunteers. Regional differences in velocities and their timings may be easily assessed and complicated motion patterns rapidly visualized.

The high temporal resolution allowed consistent visualisation of fine features of motion, while high spatial resolution allowed the detection of statistically significant regional and transmural differences in motion. Figure 1 shows example data and the mean±SD for the main velocity peaks throughout the cardiac cycle. The SDs of TTP values is small for the early systolic peaks (18.0ms for mid radial velocities, for example) but increases for the corresponding early diastolic (68.5ms) and late diastolic (129.3ms) peaks due to heart-rate variations in the healthy subject cohort (mean RR-interval = 994 +/- 121ms). Normalising to a fixed systolic and diastolic length reduces this inter-subject variation (16.1ms, 20.2ms and 29.9ms for early systolic, early diastolic and late diastolic TTPs respectively). Figure 2 shows basal, mid and apical short-axis colour-maps displaying regional velocities against time after the R-wave, averaged over the 10 volunteers.

## Conclusions

Spiral imaging has allowed the acquisition of high resolution PVM images in a relatively short acquisition time. Retrospective gating has enabled the analysis of late diastole (atrial systole). The colour plots allow easy interpretation of complicated regional motion patterns. Future work will include implementing parallel imaging to further speed up the acquisition.

## Funding

The authors acknowledge the support of Heart Research UK, Imperial College London and NIHR Royal Brompton Cardiovascular Biomedical Research Unit.
